# Use of multiplex PCR in pleural effusion: is it necessary to change the paradigm of culture-based methods?

**DOI:** 10.1099/acmi.0.000612.v3

**Published:** 2023-11-15

**Authors:** Juan Pablo Londono-Ruiz, Andrea Vargas-Garcia, Adriana Cardenas-Muller, Ana Maria Bejarano-Quintero, Maria Ximena Mantilla, Ivan Felipe Gutierrez-Tobar

**Affiliations:** ^1^​ Pediatrics Infectious Diseases, Clinica Infantil Colsubsidio, Bogotá, Colombia; ^2^​ Pediatrics Infectious Diseases, Clinica Infantil Santa Maria del Lago, Bogotá, Colombia; ^3^​ Pediatrics Infectious Diseases Fellow, Instituto Nacional de Pediatria, Ciudad de Mexico, Mexico; ^4^​ Pediatric Intensive Care Unit, Clinica Infantil Colsubsidio, Bogotá, Colombia; ^5^​ Microbiology Laboratory, Clinica Infantil Colsubsidio, Bogotá, Colombia

**Keywords:** children, multiplex PCR, pleural effusion, pleural empyema, pneumonia

## Abstract

The microbiological diagnosis of pleural effusion is based largely on classical microbiology methods, but these methods have a high rate of false negative results. Some previous studies have shown improved diagnostic performance for pathogens such as *Streptococcus pneumoniae* using molecular biology methods. We present the use of a multiplex PCR platform (BIOFIRE FILMARRAY Pneumonia Panel) for the aetiological diagnosis of pleural effusion in paediatric pneumonia. We present a case series of 17 pleural fluid samples that were processed by culture-based microbiology and molecular biology methods. Microbiological isolation was successful in four cases (25 %) through traditional culture methods. In contrast, the molecular biology panels allowed for detection in 16 out of 17 cases (94 %). The results from these panels led to a change in management for nine out of the 17 cases (52 %). This study found an increase in aetiological diagnosis in complicated pneumonia in children by using molecular biology methods, which led to a significant change in patient management.

## Data Summary

No data were generated during this research or are required for the work to be reproduced.

## Introduction

The microbiological diagnosis of pleural effusion (PE) is based largely on methods of classical microbiology. Culture-based methods allow for testing of bacterial susceptibility, but these methods take time and frequently have false negative results related to previous antimicrobial administration, among others [1]. Some studies have reported an improvement in microbiological diagnosis, especially for *Streptococcus pneumoniae,* when using PCR and culture methods versus only culture methods [2]. In a case series of adults, the multiplex PCR assay identified pathogens more frequently than conventional methods [23.3 % (7/30) vs. 6.7 % (2/30), *P*=0.008] [3]. A paediatric case series using the BioFire Film Array Blood Culture Identification (BCID) Panel (BioFire Diagnostics) on pleural fluid samples, presented as Meeting Abstracts in 2020, revealed that 10 samples were positive with the BCID panel (eight for *Streptococcus pneumoniae *, one for *Staphylococcus aureus* and one for *Streptococcus pyogenes *), compared to only 2/10 positive cultures [4]. Other studies have used the BCID panel in different sterile body fluids, including PE, although there are currently no large studies that support its use in these fluids [5, 6].

In Colombia, recent reports have documented an increase in complications related to *Streptococcus pneumoniae* pneumonia due to serotype replacement [7] and circulation of more aggressive adenoviruses in children (including more pleural effusion cases) [8]. According to the literature, *Pneumococcus* has been reported more frequently in samples sent for PCR than in samples sent for culture [2, 9]. Adenovirus cannot be identified in pleural fluid using conventional microbiology techniques. Therefore, molecular biology techniques are important to improve aetiological diagnosis for these samples.

Here we describe the utility of multiplex PCR in pleural fluid for the diagnosis of complicated pneumonia in children by using a commercial platform for respiratory samples. We chose to utilize this platform because it is the most commonly used in the country and is the primary one employed by most hospitals with molecular biology capabilities.

## Methods

### Study population

We conducted a retrospective, descriptive, case series of children with pneumonia complicated by PE, empyema or necrotizing pneumonia who underwent surgery. Pleural fluid was sent to the laboratory for culture and cytochemical testing analysis between 2021 and 2022 in a reference children’s hospital in Bogotá, Colombia. The paediatric infectious diseases (ID) and paediatric intensive care unit (PICU) team determined in which cases to use the surplus of pleural fluid to test multiplex PCR.

### Microbiology and multiplex PCR

All pleural fluid samples were analysed for conventional cultures and cytochemical testing. We cultured samples in sheep blood agar, chocolate agar and MacConkey agar (bioMérieux). We reported growth every 24 h for at least 4 days (all cultures were taken with sterile techniques during surgery). If any growth was detected, the colony was identified and tested for antimicrobial resistance using the BD Phoenix 100 system (Becton Dickinson).

We used the BIOFIRE FILMARRAY Pneumonia Panel (BioFire Diagnostics). The panel is a multiplex PCR device with 27 common lower respiratory tract infection pathogens approved by the FDA for use in sputum (including endotracheal aspirate) and bronchoalveolar lavage (including mini-BAL) sample types. The panel includes 18 bacterial pathogens, nine viral pathogens and seven antibiotic resistance genes. We took 200 µl of pleural fluid and processed the sample following the same manufacturer’s recommendations for respiratory samples. All samples were processed promptly upon arrival at the laboratory, at room temperature, within 1 h of their arrival. The results were analysed by a multidisciplinary team that included: two paediatric infectious disease physicians, paediatric critical care physicians and microbiology experts. Any change in a patient’s therapeutics was decided by this group.

### Statistical methods

We used R version 4.2.2 (The R Foundation for Statistical Computing Platform) to analyse and tabulate the information. We describe all variables in this study using descriptive statistics.

## Results

We identified 17 samples that were processed for multiplex PCR from 16 patients (Table 1). One patient was taken twice for surgery and sample collection due to two PE events. The median age was 3 years (interquartile range 2–4 years) and 5/16 (31.2 %) were female patients. Fever and respiratory distress were common signs in 14 of 16 patients (87.5 %). Pleural empyema was the most common presentation (7/17, 41 %), followed by free PE (6/17, 35 %) and necrotizing pneumonia with empyema (4/17, 23.5 %). We found viral infection, prior to the appearance of a complicated pneumonia episode, in nine of 16 patients (52.8 %), adenovirus in three cases, rhinovirus/enterovirus in two, parainfluenza virus 3 in two, and parainfluenza 2 and 4 in one. All samples were collected by video-assisted thoracoscopic surgery (VATS). Blood and pleural fluid cultures were done for all patients.

**Table 1.** Clinical and laboratory characteristics

Test	Age (years)	Gender	Diagnosis	PCR results	Culture detected (B: blood culture, P: pleural effusion culture)	Antimicrobial therapy	Change in therapy with PCR results
1	2	F	Necrotizing pneumonia wih empyema	Negative	No	Ceftriaxone	No changes
2	3	M	Free pleural effusion	Human rhinovirus/enterovirus	No	Ceftriaxone+clindamycin	No changes
3	1	F	Pleural empyema	*Streptococcus pneumoniae, Enterobacter cloacae*	No	Ceftriaxone+clindamycin	Yes, spectrum increase
4	3	M	Free pleural effusion	*Streptococcus pneumoniae, Pseudomonas aeruginosa*	No	Ceftriaxone	No changes
5	2	M	Pleural empyema	*Streptococcus pneumoniae, Haemophilus influenzae*	No	Ceftriaxone+clindamycin	No changes
6	4	M	Necrotizing pneumonia wih empyema	* Streptococcus pneumoniae *	* Streptococcus pneumoniae* (B)	Ceftriaxone+vancomycin	No changes
7	3	F	Pleural empyema	* Streptococcus pneumoniae *	* Streptococcus pneumoniae* (P)	Ceftriaxone	No changes
8	3	M	Pleural empyema	* Streptococcus pneumoniae *	No	Ceftriaxone+clindamycin	Yes, spectrum decrease
9	4	M	Pleural empyema	* Streptococcus pneumoniae *, adenovirus	No	Ceftriaxone+clindamycin	Yes, spectrum decrease
10	4	M	Free pleural effusion	* Haemophilus influenzae *, adenovirus	No	Ceftriaxone+clindamycin	Yes, spectrum increase
11	4	M	Pleural empyema	* Streptococcus pneumoniae *, human metapneumovirus	* Streptococcus pneumoniae* (B)	Ceftriaxone+clindamycin	Yes, spectrum increase
12	1	M	Necrotizing pneumonia, with empyema	* Streptococcus pneumoniae *	No	Ceftriaxone	Yes, spectrum increase
13	4	F	Free pleural effusion	Adenovirus	No	Ceftriaxone	Yes, spectrum decrease
14	2	M	Free pleural effusion	Adenovirus	No	Penicillin G	No changes
15	3	F	Free pleural effusion	Adenovirus	No	Penicillin G	Yes, spectrum decrease
16	2	M	Pleural empyema	* Streptococcus pneumoniae *, adenovirus	* Streptococcus pneumoniae* (B), *Escherichia coli* (P)	Meropenem+vancomycin	Yes, spectrum increase
17	2	M	Necrotizing pneumonia, with empyema	* Streptococcus pneumoniae *, adenovirus	No	Ceftriaxone+clindamycin	No changes

Pathogens were documented in 16 out of 17 (94 %) molecular panels performed. *Streptococcus pneumoniae* was detected in 11 out of 17 (64.7 %) multiplex PCR tests, adenovirus was detected in seven out of 17 (41.1 %) and *Haemophilus influenzae* in two out of 17 (11.7 %) tests. Other pathogens were detected in one test each, such as rhinovirus/enterovirus, human metapneumovirus, *Enterobacter cloacae* and *Pseudomonas aeruginosa *. No resistance genes were identified by PCR. Regarding pleural fluid and blood cultures, they were positive in four (25 %) patients (three patients with positive blood cultures and two with pleural fluid isolation). *Streptococcus pneumoniae* was isolated in four patients (23.5 %), of which only one was in pleural fluid. The results of semiquantitative PCR were assessed in each case. Nonetheless, given the sterility of the liquid, any detection was regarded as significant.

We found that the use of multiple PCR allowed for some changes in antibiotic management in nine out of 17 (52.9 %) of the patients. In five patients there was an increase in the antibiotic spectrum, and in four patients there was a decrease in the antibiotic spectrum.

## Discussion

The performance of culture-based microbiology tests for the aetiological diagnosis of pneumonia with PE is low [1, 9]. In paediatrics, *Streptococcus pneumoniae* and viruses are a very important cause of PE, the latter being mainly related to small and less-symptomatic effusions. Other bacteria such as *Staphylococcus aureus *, *Streptococcus pyogenes* and *H. influenzae* are also important causes of PE. Previous studies have documented that the recovery of *Streptococcus pneumoniae* in culture is difficult; it has been reported with 24 % positivity in conventional cultures compared to 71 % with PCR-based techniques [2]. This case series found an increase in pathogen detection (23.5 % in cultures vs. 94 % in multiplex PCR tests). We also documented three times more diagnoses of pneumococcus using PCR than using cultures in complicated pneumonia with PE.

Previous studies have shown that 31 % of patients with adenovirus pneumonia can be complicated by PE, this being much more frequent in patients with a severe presentation (up to 79 %) [10]. This study was carried out during a peak of severe adenoviral infections in Colombia [8]. We detected adenovirus in 41 % of the samples, of which in almost half no related bacterial pathogen was documented. In addition, all these patients with adenoviral infection without documented coinfection presented as free PE. This study also documented detection of virus in 52 % of PE samples, emphasizing the importance of these viruses in some cases of complicated pneumonia in paediatrics. A case-by-case analysis is required to determine if there are pathogens related to the PE beyond possible bacterial coinfections. In most detections of adenovirus in PE without detection of other copathogens (with PCR and culture techniques, and no other focus that could explain the presence of *Staphylococcus aureus *), clindamycin was stopped and although ceftriaxone was maintained, short courses of treatment were used in selected cases according to ID and PICU team analysis.

This case series indicates the importance of implementing PCR-based technologies for the diagnosis of PE in paediatrics and its implications as part of an Antimicrobial Stewardship Programme. Similar to previous studies [1, 2], it describes the usefulness for the diagnosis of PE especially due to *Pneumococcus*, the most frequent cause of complicated pneumonia in children. Interestingly, it also improved virus identification previously not possible with available tests to the clinicians. It is essential to analyse the results in relation to the clinical situation of the patient, as there could be false positives related to contamination. A standardized protocol that avoids sample contamination with other unrelated micro-organisms is necessary. Additional robust studies are needed to validate the use of existing platforms on the market, such as the one used in this study. For this initial descriptive study, we did not employ positive or negative controls for the panels used. Nevertheless, all samples that tested positive for *Streptococcus pneumoniae* in conventional culture also tested positive in the multiplex PCR. In the case of patient 1, who had no growth detected in the culture, no detection was observed in the multiplex PCR either. However, it is our hope that in a much larger study, much stricter controls can be implemented.

Although the use of this multiplex PCR device is not approved by the FDA for use in pleural fluid samples and its use in PE is considered off-label, this case series describes its potential usefulness and importance in contexts where there is no other approved PCR technology for diagnosis in pleural fluid samples. This could enhance the prospects of achieving a timely diagnosis and improving antibiotic therapy for paediatric patients with complicated pneumonia
